# Improved diagnosis of rheumatoid arthritis using an artificial neural network

**DOI:** 10.1038/s41598-022-13750-9

**Published:** 2022-06-13

**Authors:** Linlu Bai, Yuan Zhang, Pan Wang, Xiaojun Zhu, Jing-Wei Xiong, Liyan Cui

**Affiliations:** 1 Beijing Key Laboratory of Cardiometabolic Molecular Medicine, Institute of Molecular Medicine, College of Future Technology, Academy for Advanced Interdisciplinary Studies, and State Key Laboratory of Natural and Biomimetic Drugs, Peking University, No. 5 Yiheyuan Road, Haidian District, Beijing, 100871 China; 2 Department of Laboratory Medicine, Peking University Third Hospital, No. 49 North Garden Road, Haidian District, Beijing, 100191 China

**Keywords:** Computational biology and bioinformatics, Biomarkers

## Abstract

Rheumatoid arthritis (RA) is chronic systemic disease that can cause joint damage, disability and destructive polyarthritis. Current diagnosis of RA is based on a combination of clinical and laboratory features. However, RA diagnosis can be difficult at its disease onset on account of overlapping symptoms with other arthritis, so early recognition and diagnosis of RA permit the better management of patients. In order to improve the medical diagnosis of RA and evaluate the effects of different clinical features on RA diagnosis, we applied an artificial neural network (ANN) as the training algorithm, and used fivefold cross-validation to evaluate its performance. From each sample, we obtained data on 6 features: age, sex, rheumatoid factor, anti-citrullinated peptide antibody (CCP), 14-3-3η, and anti-carbamylated protein (CarP) antibodies. After training, this ANN model assigned each sample a probability for being either an RA patient or a non-RA patient. On the validation dataset, the F1 for all samples by this ANN model was 0.916, which was higher than the 0.906 we previously reported using an optimal threshold algorithm. Therefore, this ANN algorithm not only improved the accuracy of RA diagnosis, but also revealed that anti-CCP had the greatest effect while age and anti-CarP had a weaker on RA diagnosis.

## Introduction

Rheumatoid arthritis (RA), a chronic multisystem autoimmune disease, is caused by persistent inflammatory synovitis and subsequent erosion of joint structures. The etiology of this complex disease consists of both genetic and environmental risk factors^1^. RA is generally diagnosed based on two laboratory indicators: rheumatoid factor (RF) and anti-cyclic citrullinated peptide (CCP) antibody. However, even if these indicators are negative, a patient may still develop RA. At the same time, if one of the indicators is positive, a patient may not suffer from RA.

In a previous study, we showed that in the Han population of Northern China, anti-CarP and 14-3-3η protein are valuable indicators of RA, and when combined with RF and anti-CCP, the detection accuracy is maximized^2^. However, in the process of diagnosis, in addition to the above two indicators, other factors such as age and gender are ignored. Moreover, rheumatologists routinely use the 2010 American College of Rheumatology (ACR)/European Union of Rheumatology (EULAR) classification criteria for diagnosis, but some RA cases do not meet the criteria^3^. Therefore, we are actively working on finding more effective means and various clinical indicators to further improve the accuracy of RA diagnosis.

In recent years, artificial intelligence (AI) has made great breakthroughs in variety of scientific areas. Computer programs perform better than humans in the interpretation of medical images in clinical settings^4^. Deep learning is a sub-discipline of AI, and its application to medical image interpretation has gradually expanded. It is known that in some fields, the efficiency of computer analysis is better than that of human researchers; for example, AI is widely used to analyze magnetic resonance imaging data and predict early RA^5^. Deep learning has a wide range of applications in computer vision, and it plays an important role in analyzing imaging data of many diseases (e.g., melanoma, retinopathy, and metastatic breast cancer). A subcategory of deep learning called recurrent neural networks is the latest technology for longitudinal prediction and application in electronic health record data^6^. Integrating multiple items of data from patients to develop AI-based models has shown great potential to improve the accuracy of diagnosis, thereby resulting in clinical benefits^7^. Fukae and colleagues have transformed various kinds of clinical information from patients into two-dimensional images, and then made fine adjustments to convolutional neural networks (CNNs) to determine whether or not they have RA. This work has laid the foundation for applying deep learning to the diagnosis of RA^3^. Considering that our previous study did not include certain universal characteristics (such as age and gender)^2^, here we incorporated a deep learning ANN into our RA diagnosis and evaluated the effect of different clinical features on the outcome by re-training the network.

## Materials and methods

### Patient selection

A total of 670 participants in Peking University Third Hospital were enrolled from June 1, 2017 to May 31, 2019. They were all from the Han population in North China. The RA group contained 291 RA patients aged 17–85 years. We strictly determined RA by following the ACR 1987 diagnostic criteria^8^ and the 2010 RA classification criteria of the ACR/EULAR^9^. The interference-control group contained 223 patients diagnosed with non-RA autoimmune diseases (systemic lupus erythematosus, osteoarthritis, ulcerative colitis, ankylosing spondylitis, Hashimoto's disease, scleroderma, psoriasis, gout, vasculitis, and dermatomyositis). These non-RA patients were 18–86 years old. Each non-RA autoimmune disease with < 10 patients was combined into an "other" autoimmune disease group. The healthy controls (HC group) comprised 156 healthy individuals aged 23–74 years, which were recruited from healthy individuals undergoing routine physical examination in The Third Hospital of Peking University from June 1, 2017 to May 31, 2019. Both the interference-control and HC groups constituted the control group. The basal characteristics of study population are listed as Table 1. The study was approved by the Ethics Committee of the Third Hospital of Peking University and all methods were performed in accordance with the relevant guidelines and regulations (No. 2021-083-02). Besides, informed consent was given by all participants.

**Table 1:** Basal characteristics of study population.

	Control N = 379	RA N = 291	*P* value
Age (years)	38.49 ± 13.34	51.59 ± 15.70	< 0.001
Male (%)	242 (59.4)	225 (83.2)	< 0.001
RF (IU/mL)	121.7 ± 376.6	250.2 ± 545.4	< 0.001
Anti-CCP (U/mL)	9.81 ± 22.80	521.2 ± 725.2	< 0.001
14-3-3η (RU/mL)	0.08 ± 0.27	3.15 ± 8.68	< 0.001
Anti-CarP (ng/mL)	10.42 ± 10.86	46.60 ± 39.84	< 0.001

### Variables used in the model

Briefly, we considered 6 features (age, sex, rheumatoid factor (RF), anti-CCP, 14-3-3η, and anti-CarP) for each patient sample. RF was measured by rate-turbidimetric immunoassay using IMMAGE 800 Immunochemistry System (Beckman Coulter, USA). Anti-CCP was measured by electro-chemi-luminescence assay (ECLA) using ROCHE COBAS E601 (Roche Diagnostics GmbH, Germany). The expression level of anti-CarP and 14-3-3η in the serum samples was determined by Light Initiated Chemiluminescent Assay (LiCA) using LiCA 500 Immunoassay System (ChIVD Chemclin DiagnosticsCorp., China). All data were illustrated in accordance with the manufacturer's guidelines.

### Mathematical models

We used the open-source toolkit scikit-learn built on python to do feature engineering, model establishment, and model validation^10^. We selected the following models for evaluation: (1) Artificial Neuron Networks (with 1 or 2 hidden layers); (2) Logistic Regression; (3) Random Forest; (4) K nearest neighbors; (5) Support vector machine; (6) Gaussian Naïve Bayes; (7) Gradient boosting classifier. For each hyperparameter, we fixed the other hyperparameters, performed gradient testing, and selected the one with the best performance as the value of the hyperparameter.

### Feasibility verification

For feature selection and model selection, those performance were evaluated using fivefold cross-validation; that is, the original data were equally divided into 5 parts, and the ratios of positive and negative examples for each part were consistent with the original data sets. During each training cycle, we examined the performance of the algorithm by using 4 parts of the data as the training sets and 1 part as the test set.

For model validation, we divided the dataset into 2 parts randomly, 2/3 (447, 194 RA and 253 non-RA) for training and 1/3 (223, 97 RA and 126 non-RA) for validation. The two algorithms, threshold and ANN, are applied to the validation dataset and the performances are evaluated. These indicators are used: accuracy, area under curve (AUC), confusion matrix, F1, precision and recall.

### Feature engineering

We did feature normalization, feature selection, and feature importance evaluation for feature engineering. For normalization, we used the z-score standard scaler. The best subset selection is used for feature selection, that is, all possible subset combinations were tested and the best was selected. Based on the comparing with random false features, the feature selection was also performed by Boruta^11^. Inspired by Boruta, for the feature importance evaluation in our perceptron-based ANN model, we replaced each of the real features with the shuffled shadow features and then re-trained the model, and an importance score was given from the sum of the reduction of the accuracy and the area under curve (AUC).

### Statistical analysis

Statistical analysis was performed using GraphPad software (GraphPad Prism 8 Inc., San Diego, CA, USA). Quantitative variables were expressed either as the mean ± standard deviation or the 95% confidence interval, while categorical variables were expressed as frequency and percentage. The accuracy, area under curve (AUC), F1, precision, and recall were calculated using 2 × 2 confusion matrix. *p* < 0.05 was considered statistically significant.

## Results

### All six features play important roles in RA diagnosis

**Figure 1:**
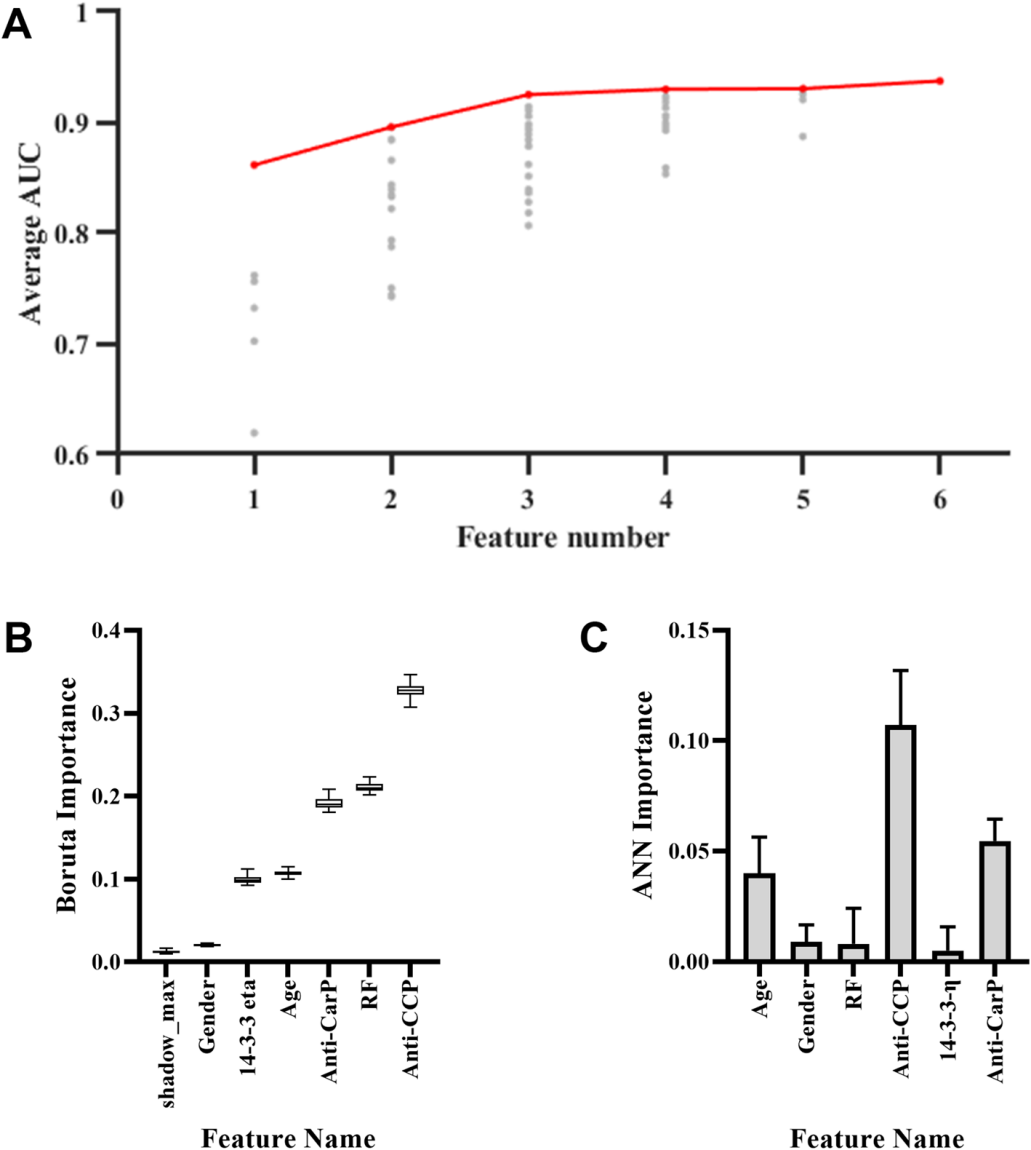
Feature selection and importance evaluation. (**A**) The model AUC increases when the number of used features increases, each grey dots indicate a combination, and the best subset of each feature number is colored red. (**B**) All features outperformed the max shadow feature in Boruta test, indicating that all features are important. The horizontal line indicates that the median and the whiskers are min to max; (**C**) In ANN model, the anti-CCP shows the most importance, and the anti-CarP and age also score high in the evaluation. Data are mean ± SEM. *RF* rheumatoid factor, *AUC* area under the curve.

To determine which of those features we will use in our model, we used the best subset selection, and tried all the combinations of the 6 features, the result is shown (Fig. 1A); each grey dots indicate a combination, and the best subset of each feature number is colored red, showing that the model AUC increases while the number of features used increases. We also used the Boruta to compare the importance of each of the feature with shadow features, and all feature hit, that is, outperform the best shadow feature, all the times as shown (Fig. 1B), indicating that all features are important. We also evaluated the feature importance in our perceptron-based ANN model (Fig. 1C), the anti-CCP showed the most importance, and the anti-CARP and age also scored high in the evaluation, showing a weaker but evident influence.

### ANN with 2 hidden layers performs best among machine learning methods

**Figure 2:**
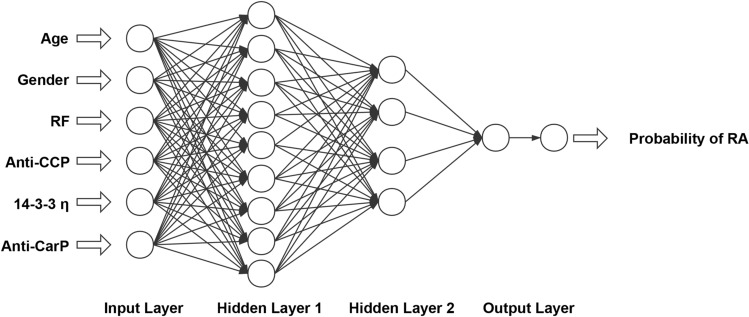
Computational structure of the artificial neural network (ANN). The inputs are age, sex, rheumatoid factor (RF), anti-CCP, 14-3-3η, and anti-CarP. This network has 2 hidden layers, one with 9 neurons and the other with 4 neurons. The output shows the probability of RA under this model.

We then tested those different machine learning models with different structures, and cross-validation results for all models were shown in Table 2, confirming that the ANN with 2 hidden layers performed best among machine learning methods. Together, with the first layer having 9 neurons and the second layer having 4 neurons (Fig. 2), the ANN gave the best result.

**Table 2:** Machine learning methods performance evaluation.

	Cross-validation accuracy (± SD)	Cross-validation AUC (± SD)
ANN (1 hidden layer)	0.901 ± 0.014	0.945 ± 0.018
ANN (2 hidden layers)	0.907 ± 0.022	0.948 ± 0.016
Logistic Regression	0.903 ± 0.013	0.947 ± 0.015
Random Forest	0.897 ± 0.019	0.937 ± 0.010
K nearest neighbors	0.879 ± 0.013	0.924 ± 0.012
Support vector machine	0.901 ± 0.014	0.890 ± 0.015
Gaussian Naïve Bayes	0.872 ± 0.020	0.942 ± 0.013
Gradient boosting classifier	0.900 ± 0.027	0.948 ± 0.009

### The ANN predicts RA diagnosis more accurately than the threshold algorithm

**Figure 3:**
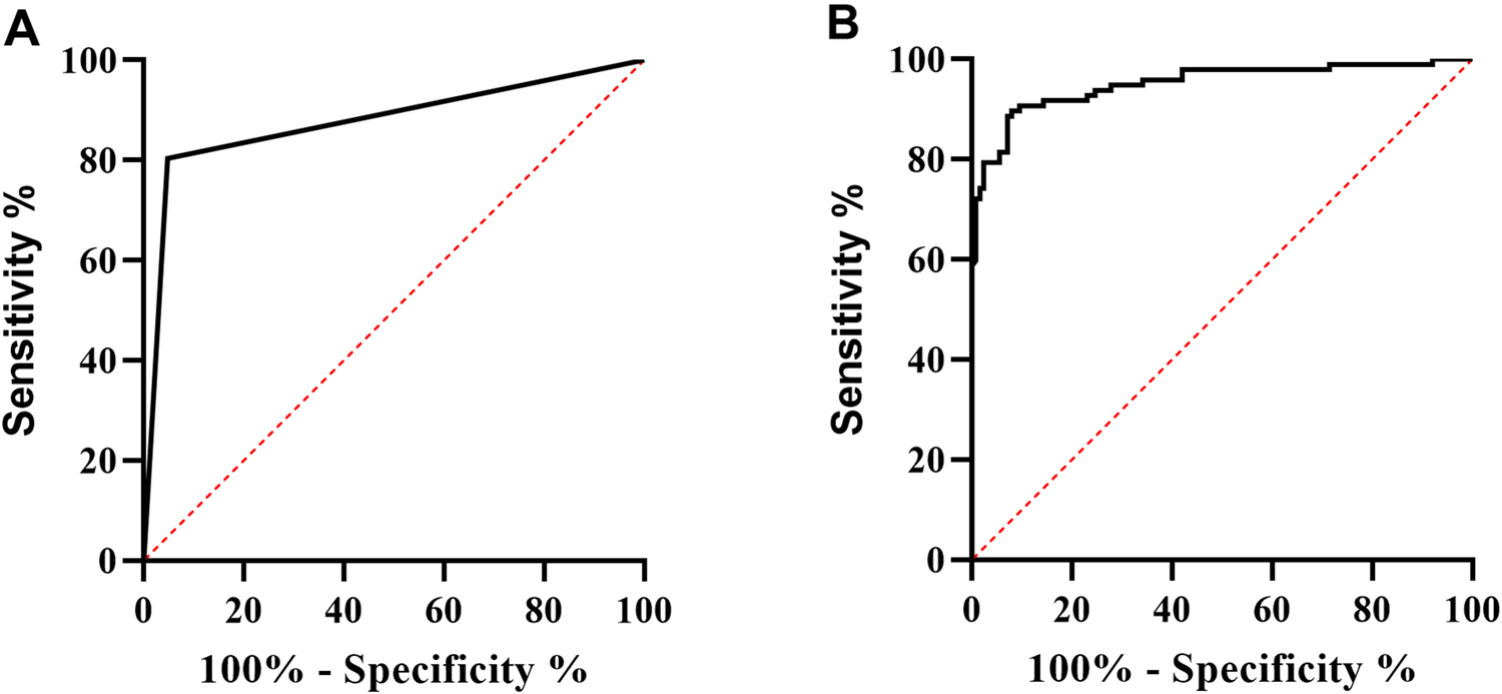
The receiver operating characteristic (ROC) curve of the previous threshold algorithm output (**A**) and the ANN output (**B**). The previous threshold algorithm with an AUC of 0.878 (95% CI: [0.826, 0.930]) and the ANN with an AUC of 0.951 (95% CI [0.921, 0.981]). *AUC* area under the curve, *CI* confidence interval.

We then asked how the ANN model performs compared with the threshold algorithm. The dataset is divided into 2 parts randomly, 2/3 (447, 194 RA and 253 non-RA) for training and 1/3 (223, 97 RA and 126 non-RA) for validation. All the evaluation was performed on the validation set. The receiver operating characteristic (ROC) curve of the ANN output is given (Fig. 3B), with an AUC of 0.951 (95% CI [0.921, 0.981]), and the ROC of the previous threshold algorithm output is also given (Fig. 3A), with an AUC of 0.878 (95% CI [0.826, 0.930]). The confusion matrixes are shown in Table 3; based on the confusion matrixes, the precision, recall, F1 and accuracy were calculated as Table 4. Though the recall of ANN method is slightly under the threshold method, the precision, F1 and accuracy overperformed the threshold method, and the AUC also indicated a satisfying classifier.

**Table 3:** Confusion matrix of threshold method and ANN method.

	Predict	
	Non-RA	RA
**Threshold**		
*Real*		
Non-RA	120	6
RA	19	78
**ANN**		
*Real*		
Non-RA	114	12
RA	9	88

**Table 4:** Comparison between the threshold and ANN methods.

	Threshold	ANN
Precision	0.863	0.927
Recall	0.952	0.905
F1	0.906	0.916
Accuracy	0.888	0.906
AUC (95%CI)	0.878([0.826,0.930])	0.951 ([0.921, 0.981])

We further asked how those mistakes happened, and the basal characteristics of 4 populations, true negative (TN), true positive (TP), false positive (FP), and false negative (FN), of which our ANN classifier are listed in Table 5. Those FN showed little signs in the traditional indicators, RF and anti-CCP, as well as showed limited sign in the new indicators, 14-3-3η and anti-CarP. Those FP shows each indicators twice over those TN. The basal characteristics of 4 populations indicates that those errors were hardly be avoid and our model accurately predicted most of the cases.

**Table 5:** Basal characteristics of 4 populations.

	TN N = 88	TP N = 114	FP N = 12	FN N = 9
Age (years)	38.08 ± 11.24	53.05 ± 14.90	52.08 ± 15.31	41.22 ± 12.73
Male (%)	66 (57.9)	72 (81.8)	9 (75.0)	6 (66.7)
RF (IU/mL)	21.7 ± 7.1	317.9 ± 584.2	44.6 ± 83.6	20.0 ± 0.0
Anti-CCP (U/mL)	7.28 ± 2.50	656.5 ± 805.8	44.7 ± 72.8	7.0 ± 0.0
14-3-3η (RU/mL)	0.06 ± 0.11	4.88 ± 13.68	0.59 ± 1.2	0.10 ± 0.22
Anti-CarP (ng/mL)	9.74 ± 9.31	58.24 ± 44.35	18.02 ± 14.66	14.12 ± 12.40

## Discussion

Technological advances in image processing and analysis have laid a solid foundation for the automatic detection and diagnosis of RA. Methods based on machine learning and deep learning can be used to automatically apply a threshold to achieve prediction by their confidence levels, so that they can be used to generate objective disease-specific RA markers of patient mobility between clinical visits^12^. In this study, we introduced an ANN into the diagnosis of RA, enabling the integration of all features to increase the accuracy of diagnosis and decrease the waste of indicator information caused by threshold division. This ANN algorithm achieved a better prediction accuracy (90.6%) than that of the threshold algorithm (88.8%)^2^. Among these features, anti-CCP had the greatest influence while age and anti-CarP also had a weaker but evident influence on RA diagnosis, allowing us to appreciate an age factor in RA diagnosis that was not previously recognized.

AI-based paradigms are useful for accurate tissue characterization and risk stratification for RA patients. In terms of Doppler ultrasound images, neural network techniques can be used in the scoring of disease activity^13^. Machine learning- and deep learning-based techniques not only automate the risk characterization process but also provide accurate cardiovascular risk stratification for the better management of RA patients^14^. A deep learning algorithm has also been used to define and analyze the specific grade of synovitis for determining the nature of arthritis^15^. Besides, others have taken advantage of pixel information from hand radiographs to design a multi-layer CNN architecture with online data augmentation, by which accuracy, sensitivity, specificity, and precision state are achieved for the diagnosis of RA^16^. The application of CNNs may reduce diagnostic effort by saving analysis time and allowing automated data screening^17^. Admittedly, the ANN is a relatively basic form of machine learning, which operates better when the feature numbers are small, but due to the small numbers, it often does not fully reflect the condition of patients. If more clinical information, such as images, symptoms, or even self-assessments, is integrated into the features, combination with other machine learning algorithms will further improve the accuracy and efficiency of the diagnosis of RA and other diseases.
